# Assessment of Selected Spatio-Temporal Gait Parameters on Subjects with Pronated Foot Posture on the Basis of Measurements Using OptoGait. A Case-Control Study

**DOI:** 10.3390/s21082805

**Published:** 2021-04-16

**Authors:** Inmaculada Requelo-Rodríguez, Aurora Castro-Méndez, Ana María Jiménez-Cebrián, María Luisa González-Elena, Inmaculada C. Palomo-Toucedo, Manuel Pabón-Carrasco

**Affiliations:** 1 Podiatry Department, University of Seville, 41009 Seville, Spain; inmarequelo@hotmail.com (I.R.-R.); maruchi1@us.es (M.L.G.-E.); ipalomo@us.es (I.C.P.-T.); 2 Department Nursing and Podiatry, Faculty of Health Sciences, Instituto de Investigación Biomédica de Málaga (IBIMA), 29071 Málaga, Spain; amjimenezc@uma.es; 3 Cruz Roja Nursing Department, University of Seville, 41009 Seville, Spain; mpabon2@us.es

**Keywords:** foot posture index, pronation, prevention, fall, risk, gait, OptoGait

## Abstract

Walking is part of daily life and in asymptomatic subjects it is relatively easy. The physiology of walking is complex and when this complex control system fails, the risk of falls increases. As a result, gait disorders have a major impact on the older adult population and have increased in frequency as a result of population aging. Therefore, the OptoGait sensor is intended to identify gait imbalances in pronating feet to try to prevent falling and injury by compensating for it with treatments that normalize such alteration. This study is intended to assess whether spatiotemporal alterations occur in the gait cycle in a young pronating population (cases) compared to a control group (non-pronating patients) analyzed with OptoGait. Method: a total of *n* = 142 participants consisting of *n* = 70 cases (pronators) and *n* = 72 healthy controls were studied by means of a 30 s treadmill program with a system of 96 OptoGait LED sensors. Results: Significant differences were found between the two groups and both feet in stride length and stride time, gait cycle duration and gait cadence (in all cases *p* < 0.05). Conclusions: pronating foot posture alters normal gait patterns measured by OptoGait; this finding presents imbalance in gait as an underlying factor. Prevention of this alteration could be considered in relation to its relationship to the risk of falling in future investigations.

## 1. Introduction

Very little research focuses on gait imbalances that present a higher a risk of causing falls [1]. On the other hand, physical exercise is vital for the elderly to maintain a healthy lifestyle. It is considered determining to identify risk factors that may alter it [2,3]. Alterations of the foot correlate with gait disturbances and instability [4]. With age, there is a reduction in osteoarticular mobility and a tendency to a more pronated posture of the foot. These changes can alter balance, functional capacity and gait patterns thus increasing the risk of falls [4].

The last decades have shown increasing attention to non-intrusive monitoring tools to analyze everyday human activities to prevent injuries in different population groups such as athletes or the elderly. A wide range of technological tools has been utilized in fall risk assessment in older adults. Novel technology can capture and analyze movement data and gait biomechanics (inertial sensors, depth camera, video, accelerometers, 3D motion tracking, Vicon, BTS Smart, Kinovea, Optogait, etc.) [1]. Some training programs have shown that gait parameters can be modified by analyzing them with the Vicon motion capture system [5]. The use of OptoGait [6,7,8] photocell sensor systems accurately quantify spatiotemporal gait patterns and analysis of their results can prevent falls by means of interventions that allow to normalize alterations involving an altered and unstable gait (custom plantar supports, rehabilitation exercises, etc.) [3,4,5,6,9]. It is argued that the use of custom foot orthoses reduces instability improves performance and gait quality [10]. Identifying early which alterations of the foot can change their kinematics is important so that it can be compensated as soon as possible and, consequently, prevent injury and risk of falling in the future [11].

The foot posture index is a validate tool to assessment a normal foot posture normal supinated or pronated. On the other hand, there is no consolidated evidence between the relationship between the position of the pronated foot and its impact on the spatiotemporal gait parameters and, consequently, a lack of coordination and risk of falling [11].

Pronated foot posture is common in adulthood, with a prevalence of around 21%. An excessive foot pronation presents an eversion in rearfoot, a dorsiflexion and an abduction of the forefoot in a static position [11,12,13,14]. This research is justified due to the important role that pronation plays as an underlying factor of fatigue, and, therefore, as a modulating factor in the prevention of spatiotemporal alterations of the gait cycle. Therefore, it is intended through the use of reliable technology to identify risk factors that from the foot may pose a risk of falling susceptible to being compensated by podiatric intervention allowing for the design of treatments that seek a more stable and safer gait [6,7,8].

Therefore, the main objective of this research was to determine whether there are alterations in the spatiotemporal gait parameters measured by the OptoGait system in subjects with a pronated foot posture index compared to healthy matched controls.

We hypothesize that patients with a pronated foot posture predispose to a modification of gait cycle parameters with respect to subjects with a normal posture index measured with the OptoGait system.

## 2. Materials and Methods

### 2.1. Trial Design

A cross-sectional case-control study; a group of subjects with a pronated foot posture (cases) is compared in relation to the spatiotemporal gait parameters evaluated with an OptoGait optical sensor system with a treadmill program lasting 30 s compared to subjects with normal foot posture (controls).

### 2.2. Participants

The total sample of *n* = 142 participants recruited from the Clinical Area of Podiatry of the University of Seville and a private podiatry clinic in Seville; *n* = 70 cases and *n* = 72 healthy controls.

The inclusion criteria: for cases were: subjects 18–65 years old with a foot posture index (FPI) in pronation >6 FPI [12]; exclusion criteria for both groups: prior diagnosis of degenerative diseases, trauma or foot/lower limb surgery that affect normal mobility or length discrepancy > 5 mm. The inclusion criteria for the control group were aged between 18 and 65 years and have a normal FPI (between 0 and +5 value).

The ethical committee of experimentation Seville evaluated this study (CD 0966-N-20); the research followed the guidelines of the Declaration of Helsinki [15], and it was registered according to the guideline of the Declaration STROBE [16].

### 2.3. Procedures

**Figure 1:**
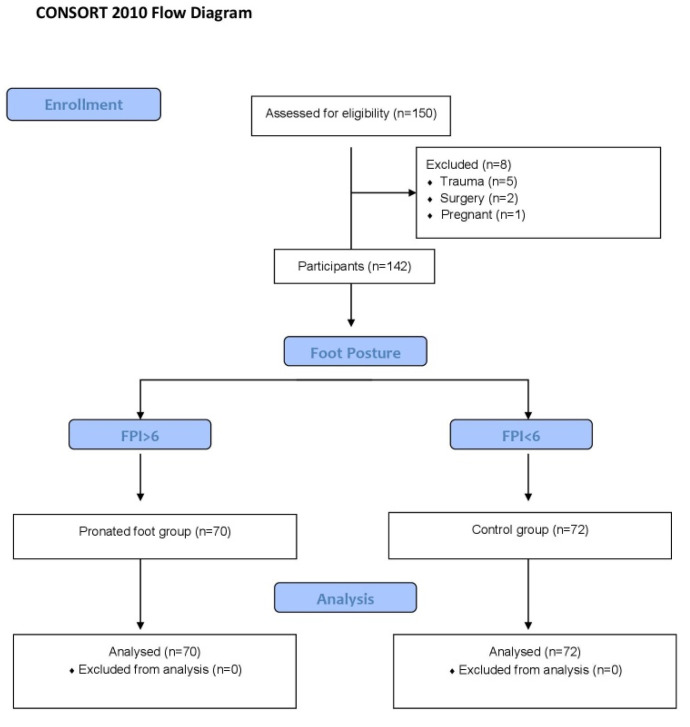
CONSORT flow diagram.

Informed consent was obtained from all subjects involved in the study, and once agreed to voluntarily participate in it, a biomechanical exploration of the feet was carried out by an experienced podiatrist (IRR) (Figure 1, CONSORT Strengt- hening the Reporting of Observational Studies). Demographic data were registered: age and gender. All participants were measured with a validate electronic weighing scale for adults and the height taken in a wall-mounter stadiometer, they were barefeet for both anthropometric data. Later, body mass index (BMI) was calculated. The foot posture index (FPI) was evaluated during the exploration, six items referring to the position of the forefoot, midfoot and rearfoot, and the three planes of motion. It is a validated and recognized method for identifying the position of the neutral, pronated or supinated foot [13].

**Figure 2:**
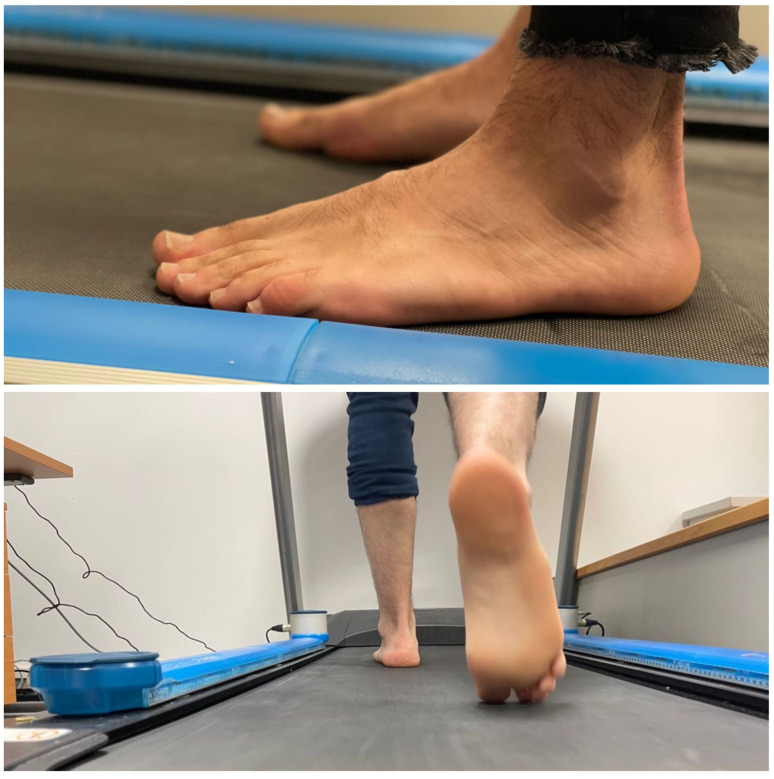
OptoGait treadmill gait analysis.

The spatiotemporal gait parameters were analyzed with OptoGait photocell sensors (Version 1.6.4.0, Microgate, Bolzano, Italy) using the predetermined treadmill program at a constant speed of 4 km per hour for 30 s according to protocol (Figure 2). The OptoGait optical data sensor system is upheld as a reliable system [6,7], providing an asymmetrical gait evaluation that allows one to evaluate the functional result before and after treatments.

A couple of one-meter photocell systems were placed next to the treadmill at a speed of 4 km per hour, and previously, the size of the subject’s bare foot was recorded. The results obtained through this system are commonly used to correct asymmetry in gait parameters by using custom plantar supports.

OptoGait results make it possible to reliably detect an alteration in the gait cycle. When the values shown by OptoGait with respect to the right and left foot differ considerably they are considered abnormal (the step length in centimeters (cm) for each foot, the stride length in seconds (sec) and percentage %). It also allows one to obtain data on the gait cycle duration in seconds and the gait cadence between subjects in steps per minute (SPM).

In this study two measurements were carried out with this sensor (performed in all cases with the subject barefoot); an initial measurement for the subject to become familiar with the device and a second measurement that was taken into account for this work. The variables collected by the system were: the step length of the right and left foot in cm, the ground contact time of the right and left foot in sec and in %, the gait cadence in steps per minute and the gait cycle duration in seconds [8,17].

This device in turn can determine the effectiveness of any treatment or training by evaluating changes in gait dysfunction over time before and after an intervention.

### 2.4. Statistical Analysis

The sample size was calculated for a power of 0.90 and an alpha error of 0.05 and a size effect of 0.5 (test family: T test, G* Power 3.0.10, Franz Faul, Kiel University, Kiel, Germany). A total sample of 70 subjects was estimated necessary for each group. A total of 144 subjects were initially recruited. In the end, 2 subjects did not comply with the research inclusion criteria.

A previous exploratory descriptive analysis was carried out: qualitative variables and quantitative variables and the distribution of the variables (Kolmogorov–Smirnov test) and the comparison between groups was done (a bivariate analysis of the qualitative variables with the chi-squared test and of the quantitative variables via the Student’s *t* test for independent groups, prior to checking the normality). The U-Mann–Whitney test was applied when necessary. In the case of a comparison between two quantitative variables, the Pearson test was used in the case of parametric samples and the Spearman test if the variable has a non-parametric behavior. All data was described as average ± standard deviation (SD). The median and interquartile range in the case of non-parametric data.

All the analyses were done using SPSS^®^ version 24.0. A *p* value of <0.05 was established as statistically significant. An intention-to-treat analysis was done.

## 3. Results

### 3.1. Description of the Total Sample and by Groups

The size of the final sample was 142 participants. The sample showed parity with respect to sex (67 males and 75 females). The average age was 26.39 ± 1.05 years (range 20–49 years old) and the body mass index (BMI) was 22.59 ± 2.84 (normal weight); (height ± SD of 174.70 ± 10.42 cm and weight 69.45 ± 2.84 kg in case group and 172.21 ± 10.63 cm and 66.25 ± 2.54 kg, in control group 172.21 ± 10.63 cm and 66.25 ± 2.54 kg).

An analysis was conducted for the variables age, sex, BMI and FPI (a validate tool to assessment the foot posture normal supinated or pronated) for each foot of the total sample, both for the case and the control groups. The data is shown below (Table 1).

Table 1 shows a significant *p*-value in the baseline value with respect to the FPI of both feet.

The sample being homogeneous with respect to sex and body mass index.

The next analysis shows the descriptive results for the gait parameters between both groups after using the OptoGait sensor. This data is presented in Table 2.

### 3.2. Statistical Analysis

The Mann–Whitney U-Test was used. Significance set at *p* < 0.05. * *p* < 0.05; ** *p* < 0.01 and *** *p* < 0.001.

The results show statistical significance for the variables: right and left stride length, right and left stride time expressed in sec and %, the gait cycle and the cadence between the two groups (in all cases *p* < 0.05).

In both groups the selected speed was the same, 4 km/h; in broad terms, the results showed that when comparing the cases with the controls the stride length of the right and left foot were significant respectively (*p* = 0.022 and *p* = 0.019), the pronating group showed an increase in length as well. With respect to the ground contact time for the right and left foot in sec and %, statistical differences were detected (*p* < 0.001 in both cases), and the gait cycle and the cadence between groups were statistically significant (*p* = 0.001 and *p* = 0.002).

The length of the stride was longer for both feet in case groups, and a longer ground contact time in seconds for both feet, the gait cycle was longer in this group as opposed to the control group where the gait cadence value represented in steps per minute was greater.

## 4. Discussion

To our knowledge, this is the first study to show new evidence of alterations in the gait cycle recorded with the OptoGait optical sensor system in a group of subjects with pronated foot posture versus subjects with a normal posture index.

Based on the results obtained in this work, statistically significant spatiotemporal modifications were present in the gait cycle measured by an OptoGait optical sensor between a group of cases and controls of the stride length of the right and left foot, right and left foot stride time in seconds, gait cycle duration and gait cadence (*p* value < 0.05).

Due to the novelty of this topic, discussion is difficult because of the inability to compare our results with previous studies using the OptoGait system. However, others researches have compared gait patterns in healthy subjects and also in participants with biomechanical alterations using other sensor systems [18,19,20]. 

One study conducted, Muchna et al. (2017), used a gait analysis system (LEGSys) and assessed the gait speed and stride length between two groups of subjects with and without biomechanical alterations of the foot. The results showed that subjects with foot alterations exhibited significant gait speed and stride length when comparing both groups [18].

Another recent study conducted by Dodelin et al. (2020) compared two groups of barefoot subjects using the Oxford Foot model motion capture system, the results concluded that the pronated feet induced modifications throughout the kinematic chain during the gait, thus increasing significantly the contact time of the hallux [20]. This data is consistent with other works, in which the pronation of the foot showed greater maximum pressure, contact area predominantly in the medial arch, central forefoot and hallux [21].

Therefore, the conclusions of these studies support our results; foot pronation alters the dynamic behavior of the gait and consequently its optimal development. In 2007, a study concluded that orthotic foot treatment improved postural control in subjects with foot-level disorders after six weeks of use [22]. This affirmation is reaffirmed by other studies done, which argue that the use of plantar supports in pronating subjects stabilizes the foot and helps with balance by limiting its pronation [23,24,25]

Based on scientific literature, foot disorders are defended in relation to the risk of falling [4,11,26]. The presence of hallux abducto valgus, plantar fasciitis or limitation of hallux or ankle range [4,18,26,27] has been proven to be underlying factors of foot pronation. In general, people who tend to fall have difficulty adjusting their unipedal posture when walking, which, consequently, alters their gait patterns.

Monitoring and gait analysis should be performed on a constant basis for early detection and stage classification of neurological diseases, such as Parkinson’s, which is linked to the risk of falling [28]. Some of these diseases in older people develop slowly (up to years of latency) and symptoms are imperceptible in the early stages; daily monitoring and long-term recording of gait characteristics is critical to accurately observing gait anomalies [28].

A large set of evidence shows that the stride length of each foot, the step length, the ground contact time of each foot and the gait cadence are key early indicators for when these gait patterns are altered compared to healthy controls [29].

Recently, Zhang et al. [30] conducted a study where they identified fatigue indicators using gait variability measures. The authors reported a significant difference in the pronation angle of the heel (*p* = 0.005) and the variability and energy consumption of the angles on the anteroposterior axis (*p* = 0.028, *p* = 0.028), medial-lateral axis (*p* = 0.014, *p* = 0.014) and vertical axis (*p* = 0.002, *p* < 0.001). The authors highlight an increase in the angle of rotation in the vertical axis and the maximum angle of rotation on the medial-lateral axis. This showed that instability due to walking long distance or fatigue could induce heel pronation. Altered alignment of the lower extremities may increase the risk of overuse injuries [31]. This could be why antipronation shoes were developed for runners with pronated feet [32]. Therefore, if a foot starts pronating at a young age it can increase over time causing an increased risk in older age.

It is therefore considered important to identify early factors, mainly in the elderly, that modify normal gait and affect its balance because foot pathology predisposes to more than twice the risk of falling, therefore, causing a very serious impact on quality of life [18,33,34,35,36]. Biomechanical alterations of the foot are related to injuries on other levels of the lower extremity and increased risk of falling. Analysis using reliable technological devices can identify alterations that induce gait instability as has been done in this study on pronating subjects [5,6]. Therefore gait may be successfully reduce or even avoid the risk of fall for the improvement of gait biomechanics patterns [5]. The foot disorders are common in older adults with a high prevalence of falls and functional limitations and gait imbalances. Foot pronation is also very prevalent in elderly people and studies seem to indicate the increasing of this condition along lifetime. Consequently, if foot and gait problems can be identified early, we can implement preventive interventions to normalize normal biomechanics of gait for example in pronated foot posture. Falls and fall-related injuries are predictable and preventable with interventions targeting modifiable risk factors such as muscle strength or custom made-foot orthoses, which improve balance and mobility.

This can be interesting from a point of view of prevention, especially in the risk of falling in the elderly by means of early compensation of the foot, such as using plantar supports, which allows for a more stable gait. These associations can be a key focus for developing new intervention strategies for the prevention of falls in patients with FPI alterations tending towards pronation.

## 5. Conclusions

The results of this study, in which evaluating spatiotemporal parameters of the gait cycle measured by an optical system of OptoGait sensors were associated significant modifications in a group of pronating subjects (cases), compared to a group with normal foot posture index (controls), relative to stride length and stride time for each foot, duration of the gait cycle and cadence.

### 5.1. Limitations

Some limitations should be considered in this study. On one hand, the age range of the sample included only young subjects; it would be interesting to conduct a similar study on elderly subjects. The different age categories could not be considered in this research. Although the initial inclusion criterion was of subjects from 18 to 65 years old, finally no significant differences were found regarding the age of the participants, so it was not possible to identify different age categories for the youth of the sample. This has been a limitation of the study.

On the other, the subjects walked at self-selected speeds. The design was cross-sectional and thus addresses associations only, not causality. This work has been presented as a preliminary cohort study of this sample. Future studies are necessary.

### 5.2. Practical Implications of the Study

Assessing foot problems and patient referrals to podiatrists should be a routine component of assessing and preventing the risk of falls.

## Figures and Tables

**Table 1:** Whole sample demographic values of the demographic variables for both groups. The foot posture index for right and left feet was showed (foot posture index (FPI)) (average ± standard deviation) are shown.

Sample *N* = 142		Group		
		Case *n* = 70	Control *n* = 72	*p*-Value
Gender Female	75 (52.8%)	38 (54.32%)	37 (51.38%)	*p* = 0.427 ^a^
BMI	22.55 (3.11)	23.00 (3.54)	22.00 (4.40)	*p* = 0.082 ^b^
FPI right foot	4.65 (3.00)	7.00 (1.00)	4.00 (4.00)	*p* = 0.001 ^b^
FPI left foot	4.30 (4.00)	7.00 (1.20)	4.00 (3.80)	*p* = 0.001 ^b^

^a^ Chi-squared test. ^b^ Mann–Whitney U-test. Values are presented as median (interquartile ranges). BMI: body mass index.

**Table 2:** Descriptive statistical analysis after the OptoGait sensor gait analysis between the control and case groups.

Sample *n* = 142 *p*-Value		Group		
		Case *n* = 70	Control *n* = 72	*p*-Value
Right foot stride length	60.70 (4.60)	62.50 (7.75)	60.40 (1.70)	*p* = 0.022
Left foot stride length	60.85 (4,48)	62.70 (5.75)	60.30 (2.75)	*p* = 0.019
Ground contact time,				
right foot sec	0.54 (0.05)	0.59 (0.06)	0.54 (0.01)	*p* = 0.001
Step %	70.90 (6.63)	71.10 (4.55)	70.50 (6.75)	*p* = 0.460
Ground contact time,				
left foot sec	0.54 (0.03)	0.57(0.06)	0.54 (0.03)	*p* = 0.001
Step %	70.60 (6.00)	70.90(5.00)	70.40 (7.40)	*p* = 0.081
Gait cycle sec	1.11 (0.09)	1.16 (0.09)	1.09 (0.05)	*p* = 0.001
Gait cadence	107.90 (8.83)	102.40 (13.20)	109.00 (3.70)	*p* = 0.002

## Data Availability

Not applicable.

## References

[1] Sun R., Sosnoff J.J. (2018). Novel sensing technology in fall risk assessment in older adults: A systematic review. BMC Geriatr..

[2] Stevens J.A. (2005). Falls among older adults—Risk factors and prevention strategies. J. Safety Res..

[3] Hanatsu N.H., Begg R.K. (2018). Shoe-Insole Technology for Injury Prevention in Walking. Sensors.

[4] Menz H.B., Maria A., Martin J.S. (2018). Foot problems as a risk factor for falls in community-dwelling older people: A systematic review and meta-analysis. Maturitas.

[5] Shengwu Y.L. (2020). Effects of Step Retraining on impact forces, lower limb biomechanics and leg stiffness. J. Biomed. Eng..

[6] Lee M., Song C., Lee K., Shin D., Shin S. (2014). Agreement between the spatio-temporal gait parameters from treadmill-based photoelectric cell and the instrumented treadmill system in healthy young adults and stroke patients. Med. Sci. Monit..

[7] Jaén-Carrillo D., García-Pinillos F., Cartón-Llorente A., Almenar-Arasanz A.J., Bustillo-Pelayo J.A., Roche-Seruendo L.E. (2020). Prueba-reprueba la confiabilidad del Sistema OptoGait para el análisis de los parámetros de la marcha en Carrera espacio-temporal y la rigidez de la parte inferior del cuerpo en adultos sanos. Actas Inst. Ing. Mecánicos Parte P Rev. Ing. Tecnol. Deport..

[8] User Manual. Microgate, Bolzano, Italia OptoGait. http://www.optogait.com/OptoGaitPortal/Media/Manuals/Manual-ES.PDF.

[9] Demirel A., Onan D., Oz M., Ozel A.Y., Ulger O. (2020). Moderate disability has negative effect on spatiotemporal parameters in patients with chronic low back pain. Gait Posture.

[10] Canuel-Laurenson É. (2020). Effect of foot orthoses on walking performance and quality in the elderly. Rev. Du Podol..

[11] Calvo-Lobo C., Painceira-Villar R., García-Paz V., Becerro-de-Bengoa-Vallejo R., Losa-Iglesias M.E., Munuera-Martínez P.V., López-López D. (2019). Falls rate increase and foot dorsal flexion limitations are exhibited in patients who suffer from asthma: A novel case-control study. Int. J. Med. Sci..

[12] Sánchez-Rodríguez R., Valle-Estévez S., Fraile-García P.A., Martínez-Nova A., Gómez-Martín B., Escamilla-Martínez E. (2020). Modification of Pronated Foot Posture after a Program of Therapeutic Exercises. Int. J. Environ. Res. Public Health.

[13] Das R., Kumar N. (2014). Investigations on postural stability and spatiotemporal parameters of human gait using developed wearable smart insole. J. Med. Eng. Technol..

[14] Redmond A.C., Crane Y.Z., Menz H.B. (2008). Normative values for the Foot Posture Index. J. Foot. Ankle Res..

[15] Declaración de Helsinki de la AMM—Principios éticos Para Las Investigaciones Médicas en Seres Humanos Asamblea General de la AMM, Fortaleza, Brasil, Octubre de 2013. https://www.wma.net/es/policies-post/declaracion-de-helsinki-de-la-amm-principios-eticos-para-las-investigaciones-medicas-en-seres-humanos/.

[16] Vandenbroucke J.P., von Elm E., Altman D.G., Gotzsche P.C., Mulrow C.D., Pocock S. (2007). Strengthening the Reporting of Observational etudies in Epidemiology (STROBE): Explanation and elaboration. Epidemiology.

[17] User Manual. Microgate, Bolzano, Italia OptoGait, Gait Parameters. http://www.optogait.com/Aplicaciones/Ritmo-optimo-de-marcha.

[18] Muchna A., Najafi B., Wendel C.S., Schwenk M., Armstrong D.G., Mohler J. (2017). Foot problems in older adults: Associations with incident falls, frailty syndrome and sensor-derived gait, balance, and physical activity measures. J. Am. Podiatr. Med. Assoc..

[19] Najafi B., Khan T., Wrobel J. Laboratory in a box: Wearable sensors and its advantages for gait analysis. Proceedings of the 2011 Annual International Conference of the IEEE Engineering in Medicine and Biology Society.

[20] Dodelin D., Tourny C., L’Hermette M. (2020). The biomechanical effects of pronated foot function on gait. An experimental study. Scand. J. Med. Sci. Sports.

[21] Buldt A.K., Allan J.J., Landorf K.B., Menz H.B. (2018). The relationship between foot posture and plantar pressure during walking in adults: A systematic review. Gait Posture.

[22] Mattacola C.G., Dwyer M.K., Miller A.K., Uhl T.L., McCrory J.L., Malone T.R. (2007). Effect of orthoses on postural stability in asymptomatic subjects with rearfoot malalignment during a 6-week acclimation period. Arch. Phys. Med. Rehabil..

[23] Rome K., Marrón C.L. (2004). Randomized clinical trial into the impact of rigid foot orthoses on balance parameters in excessively pronated feet. Reh. Clin..

[24] Gross M.T., Mercer V.S., Lin F.C. (2012). Effects of foot orthoses on balance in older adults. J. Orthop. Sports Phys. Ther..

[25] Banwell H.A., Mackintosh S., Thewlis D. (2014). Foot orthoses for adults with flexible pes planus: A systematic review. J. Foot Ankle Res..

[26] Chaiwanichsiri D., Janchai S., Tantisiriwat N. (2009). Foot Disorders and Falls in Older Persons. Gerontology.

[27] Menz H.B., Auhl M., Tan J.M., Andrew K.B., Shannon E.M. (2018). Centre of pressure characteristics during walking in individuals with and without first metatarsophalangeal joint osteoarthritis. Gait Posture.

[28] Hua R., Wang Y. (2019). Monitoring Insole (MONI): A Low Power Solution toward Daily Gait Monitoring and Analysis. IEEE Sens. J..

[29] Hollman J., McDade E., Petersen R. (2010). Normative spatiotemporal gait parameters in older adults. Gait Posture.

[30] Zhang G., Wong I.K.-K., Chen T.L.-W., Hong T.T.-H., Wong D.W.-C., Peng Y., Yan F., Wang Y., Tan Q., Zhang M. (2020). Identifying Fatigue Indicators Using Gait Variability Measures: A Longitudinal Study on Elderly Brisk Walking. Sensors.

[31] Tong J.W., Kong P.W. (2013). Association between Foot Type and Lower Extremity Injuries: Systematic Literature Review with Meta-analysis. J. Orthop. Sports Phys. Ther..

[32] Jafarnezhadgero A., Alavi-Mehr S.M., Granacher U. (2019). Effects of anti-pronation shoes on lower limb kinematics and kinetics in female runners with pronated feet: The role of physical fatigue. PLoS ONE.

[33] Thomas E., Battaglia G., Patti A., Brusa J., Leonardi V., Palma A., Bellafiore M. (2019). Physical activity programs for balance and fall prevention in elderly: A systematic review. Medicine.

[34] Global Recommendations on Physical Activity for Health. https://www.who.int/dietphysicalactivity/global-PA-recs-2010.pdf.

[35] Paterson D.H., Warburton D.E. (2010). Physical activity and functional limitations in older adults: A systematic review related to Canada’s Physical Activity Guidelines. Int. J. Behav. Nutr. Phys. Act..

[36] Menz H.B., Morris M.E., Lord S.R. (2006). Foot and ankle risk factors for falls in older people: A prospective study. J. Gerontol. A Biol. Sci. Med. Sci..

